# Point mutations in *KAL1* and the mitochondrial gene *MT-tRNA*^*cys*^ synergize to produce Kallmann syndrome phenotype

**DOI:** 10.1038/srep13050

**Published:** 2015-08-17

**Authors:** Fei Wang, Guo-dong Huang, Hui Tian, Ying-bin Zhong, Hui-juan Shi, Zheng Li, Xian-sheng Zhang, Han Wang, Fei Sun

**Affiliations:** 1 Hefei National Laboratory for Physical Sciences at Microscale and School of Life Sciences, University of Science and Technology of China, Hefei, Anhui 230026, China; 2 Center for Circadian Clocks, Medical College, Soochow University, Suzhou 215123, Jiangsu, China; 3 School of Biology & Basic Medical Sciences, Medical College, Soochow University, Suzhou 215123, Jiangsu, China; 4 National Population and Family Planning Key Laboratory of Contraceptive Drugs and Devices, Shanghai Institute of Planned Parenthood Research, Shanghai, China; 5 Department of Urology, Shanghai Human Sperm Bank, Renji Hospital, Shanghai Jiao Tong University School of Medicine, Shanghai, 200127, China; 6 Departments of Urology, the First Affiliated Hospital of Anhui Medical University, Hefei, Anhui 230032, China

## Abstract

Kallmann syndrome (KS) is an inherited developmental disorder defined as the association of hypogonadotropic hypogonadism and anosmia or hyposmia. KS has been shown to be a genetically heterogeneous disease with different modes of inheritance. However, variants in any of the causative genes identified so far are only found in approximately one third of KS patients, thus indicating that other genes or pathways remain to be discovered. Here, we report a large Han Chinese family with inherited KS which harbors two novel variants, *KAL1* c.146G>T (p.Cys49Phe) and mitochondrial *tRNA*^*cys*^ (m.5800A>G). Although two variants can’t exert obvious effects on the migration of GnRH neurons, they show the synergistic effect, which can account for the occurrence of the disorder in this family. Furthermore, the disturbance of the mitochondrial cysteinyl-tRNA pathway can significantly affect the migration of GnRH cells *in vitro* and *in vivo* by influencing the chemomigration function of anosmin-1. Our work highlights a new mode of inheritance underlay the genetic etiology of KS and provide valuable clues to understand the disease development.

Kallmann syndrome (KS [MIM 147950, 244200, 308700, 610628, 612370, and 612702]) is defined by the occurrence of congenital hypogonadotropic hypogonadism (CHH) and anosmia/hyposmia due to gonadotropin-releasing hormone (GnRH) deficiency and the abnormal development of the peripheral olfactory system (olfactory nerves and olfactory bulbs). During normal embryonic development, the olfactory neurons project their axons to the olfactory bulb through the cribriform plate and the meningeal tissue, while GnRH neurons migrate along the pathway of the olfactory nerve fibers from the nose to the brain^1^,^2^. Premature interruption of the olfactory, vomeronasal, and terminal nerve fibers in the frontonasal region disrupts the migration of the GnRH cells^3^.

CHH disorders are characterized by delayed or absent puberty, infertility, and low plasma levels of gonadotropins and, consequently, gonadal steroids^4^. KS accounts for approximately 40% of the total CHH cases and is generally considered to compose a distinct subgroup^5^. The prevalence of KS has been roughly estimated at 1 in 8000 males and 1 in 40,000 females, but this rate may be underestimated, especially in females^6^. Although most KS patients present as sporadic cases, many cases are clearly familial. The gene underlay the X-linked form of KS, *KAL1* (OMIM: 308700; NM_000216.2), which encodes the extracellular matrix glycoprotein anosmin-1, is first identified in 1991^7^,^8^. Studies have demonstrated that KS is a genetically heterogeneous disease with different modes of transmission, including X-linked recessive, autosomal recessive, autosomal dominant with incomplete penetrance, and most likely, digenic/oligogenic inheritance^6^. Variants in the genes encoding fibroblast growth factor receptor 1 (FGFR1) and fibroblast growth factor 8 (FGF8) have been shown to cause CHH^9^,^10^,^11^, leading to the identification of the critical role of fibroblast growth factor (FGF) signaling in olfactory placode induction, differentiation, and GnRH neuronal fate specification^12^. Anosmin-1, along with heparin sulfate (HS) modified with specific 6-O-sulfates, can interact with FGFR1 and modulate FGF signaling^13^,^14^. Other responsible genes that are involved in FGFR1 signaling and are mutated in CHH/KS patients remain to be discovered. Using protein-protein interactome data to identify high-quality candidate genes, variants in genes in the “FGF8 synexpression” group, including *FGF17*, *IL17RD*, *DUSP6*, *SPRY4*, and *FLRT3*, were identified in individuals with CHH^15^. In addition to proteins involved in FGF signaling, prokineticin2 (PROK2), prokineticin receptor 2 (PROKR2)^16^,^17^, GNRH1, GNRHR1^18^, KISS1R^19^, TAC3, TACR3^20^, CHD7^21^, and WDR11^22^ have also been shown to be mutated in CHH/KS patients. Furthermore, digenic or oligogenic mode is another feature of CHH/KS inheritance^23^,^24^,^25^,^26^,^27^.

However, mutations in any of these genes have been identified in no more than one third of KS individuals^6^. The infertility of affected individuals and the complex mode of disease inheritance impede positional cloning strategies using linkage analysis^16^. Other strategies have been used to identify the causative genes, including the analysis of rare KS individuals who carry chromosomal rearrangements that are detectable by cytogenetic techniques^16^. Using a pan-genomic approach, *SEMA3A* was identified as a new gene, whose loss-of-function is involved in KS^28^. Incidentally, *SOX10* mutations have been found to be associated with olfactory bulb agenesis and cause KS^29^. However, these newly identified genes can only be responsible for a small portion of KS patients; for example, *SOX10* mutations are rare in KS individuals without hearing impairments^29^.

Here, we described a large Han Chinese family with inherited KS. In this family, the *KAL1* gene harbored a rare c.146G>T variant (p.Cys49Phe), which was not shown to have obvious deleterious effects on the protein function. However, analysis of the mitochondrial genome of the matrilineal lineage identified a novel, nearly homoplasmic variant resulting in the substitution of a guanine residue for an adenine residue located adjacent to the 5′ region of the mitochondrial tRNA^cys^ (*MT-tRNA*^*cys*^) anticodon. The synergistic effect of the variants in anosmin-1 and MT-tRNA^cys^ caused the abnormal migration of GnRH cells. We also showed that perturbance in the mitochondrial cysteinyl-tRNA pathway could significantly affect the migration of GnRH cells *in vitro* and *in vivo*. These results highlighted the mitochondrial involvement in the migration of GnRH cells. Given the genetic heterogeneity of KS, these findings shed new light on the genetic etiology of these developmental disorders.

## Results

### One large Han Chinese family with KS

**Figure 1:**
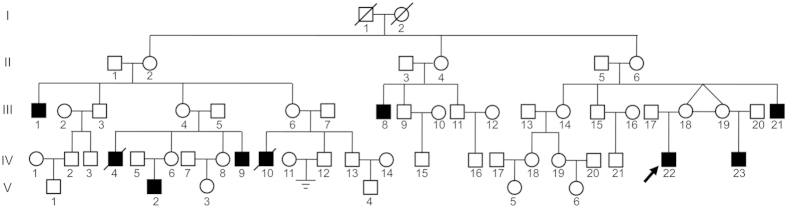
The pedigree of a five-generation Han Chinese family with KS. The men are indicated by squares, and the women are indicated by circles. The symbols marked by a slash indicate that the subject is deceased. Black symbols denote the affected individuals, while the white symbols denote the unaffected individuals. The black arrow denotes the proband.

A non-consanguineous Han Chinese male (Subject IV-22) was diagnosed with KS, who showed sexual infantilism and male infertility. He was a 29-year-old man, with a height of almost 170 cm, a weight of 55 kg, and an arm span of 173 cm. Puberty had not occurred by the age of 18 years old, even though he had received hCG hormone substitutions for many years. Physical examination showed a hypogonadal aspect, with absent facial hair, sparse pubic hair (Tanner stage 2), and a 4-cm penis. He had bilateral scrotal testes with volumes of 2 and 3 ml (normal for age, 15 to 30 ml). Gynecomastia was absent. A clinical smell test revealed anosmia. The karyotype was 46, XY. As indicated in Supp. Table S1, his serum testosterone concentration was 0.34 nmol/L (normal rage, 9.9 to 27.8 nmol/L), and his basal serum luteinizing hormone (LH) and follicle-stimulating hormone (FSH) concentrations were 0.1 (normal range, 1.7 to 8.6) and 0.88 (normal range, 1.5 to 12.4) IU/L, respectively. The results for the levels of growth hormone, pituitary prolactin, as well as thyroid and adrenal pituitary functions and glycolipid metabolism, were normal. He had no spontaneous spermatorrhea and was unable to masturbate to ejaculation. The evaluation of his extended family revealed a high prevalence of these KS males in the matrilineal lineage, while all of the female members were normal (Fig. 1). We ultimately performed a detailed clinical evaluation of all available relatives in the family (Supp. Table S1). Including the index case, 9 family members had CHH with complete or incomplete olfaction disturbance. None of the patients had any other phenotypic abnormalities, e.g., cleft lip, abnormal eye movements, hearing loss, abnormalities of tooth development, unilateral renal agenesis and bimanual synkinesis, except for similar symptoms related to hypogonadism to some extent, e.g., microrchidia, secondary sexual characteristics growth retardation and male infertility; two patients had died by the time of the investigation, and their phenotypes were described by their relatives. The older patients (subject III-1, III-8, III-21) suffered from metabolic syndromes due to testosterone deficiency.

### The *KAL1* gene harbored a rare sequence variant that was closely linked to the KS phenotype but could not be shown to alter the structure or function of anosmin-1

**Figure 2:**
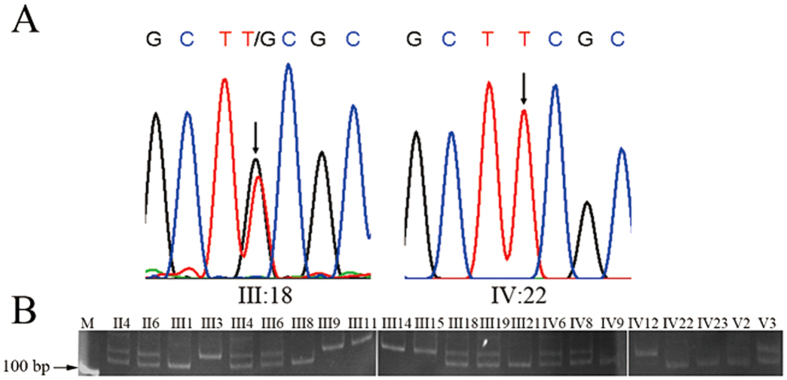
The *KAL1* gene harbored a rare variant c.146G>T (p.Cys49Phe) in the KS individuals. (**A**) Exon Sequencing confirmed the *KAL1* c.146G>T variant. (**B**) The genotypes of the family members as determined by PCR-RFLP.

**Figure 3:**
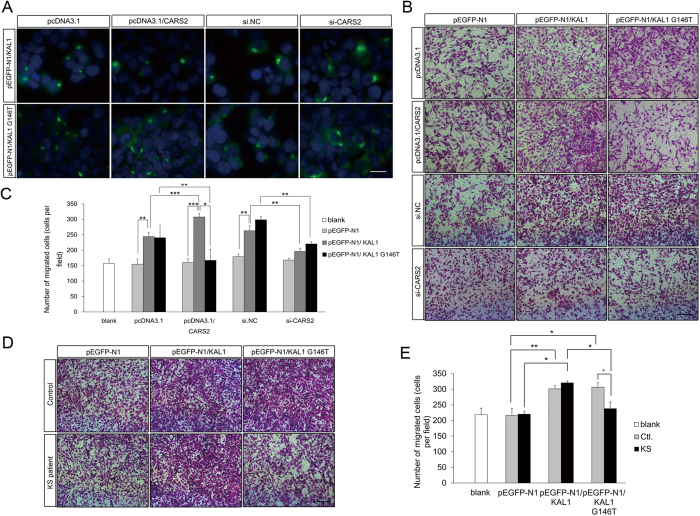
Perturbance in the mitochondrial cysteinyl-tRNA pathway synergize with anosmin-1 variant to affect the migration of NLT cells *in vitro*. (**A**) Both the wild-type and c.146G>T mutated anosmin-1-GFP were aggregated or dispersed after overexpression or knockdown of CARS2, respectively. Blue, Hoechst; green, anosmin-1-GFP. Scale bar: 50 μm. (**B**,**C**) Chemomigration of the NLT neurons exposed to different media from 293T cells transfected with the anosmin-1 wild-type/mutant and/or CARS2 overexpression vectors/siRNAs. The migrated NLT cells were captured after exposure to different groups of medium for 24 h (**B**), and the cell number was counted (**C**). No differences in cell migration were observed when cells were treated with the media containing wild-type or mutant anosmin-1. Overexpression of CARS2 increased the wild-type anosmin-1-induced migration, but it decreased the c.146G>T mutated anosmin-1-induced NLT migration. Scale bar: 50 μm. (**D**,**E**) NLT neuron migration was measured after exposure to media from control and KS B cell lines transfected with the wild-type/mutated anosmin-1. The number of migrated cells was decreased after incubation in medium from B cell lines of KS patients transfected mutated anosmin-1. Scale bar: 50 μm. The data are presented as the mean ± S.E.M. for at least three independent experiments. Statistical analysis by ANOVA: *p < 0.05, **p < 0.01, ***p < 0.001.

The X-linked recessive mode was the most likely Mendelian inheritance pattern in the pedigree, and detailed genetic analysis by STR genotyping also showed that fragments of the X chromosome region Xp22.32 were closely linked to the KS phenotype (Supp. Figure S1). Other candidate genes (*FGFR1, PROK2, PROK2R and FGF8*), also had been excluded by clinical lab (Data not shown). At present, *KAL1* is the only known KS candidate gene located in X chromosome which variants show X-linked recessive inheritance. So, all coding exons and splice junctions of *KAL1* gene were directly sequenced for variations. A novel, non-synonymous c.146G>T variant (p.Cys49Phe) was identified (Fig. 2A,B and Supp. Figure S2), and this variant was absent from the databases (dbSNP, 1000 Genome Project, NHLBI Exome Variant Server and human mitochondrial database) and by screening at least 2,000 control samples from general population that we tested. However, the non-synonymous amino acid substitution in anosmin-1 did not significantly disrupted the protein structure, predicted by the modeling tool, Phyre2 (Supplementary data). The mutated cysteine residue was also not predicted to be involved in a disulphide bond^3^. Moreover, when GFP-tagged wild-type and mutated anosmin-1 were expressed in 293T cells, they were all highly expressed, accumulated in the endoplasmic reticulum (ER) and in the Golgi bodies (Fig. 3A, Supp. Figure S4A and S4B) and secreted to the media with similar level (Supp. Figure S4C and S4D). Thus, it was demonstrated that the structural characteristics and the localization of anosmin-1 were not affected by the variation. It has been reported that anosmin-1 increases the chemomigration of the GnRH neuronal GN11 cells^30^. To further confirm the roles of wild-type and mutated anosmin-1 in the induction of GnRH neuron migration, transwell migration assays were performed using NLT cells, which is a mouse GnRH cell line^30^, by incubating the chamber in control media or media from pEGFP-KAL1 or pEGFP-KAL1 G146T-transfected 293T cells. The results showed that the exposure of the NLT cells to media enriched in anosmin-1 strongly induced the migration of the cells (Fig. 3B,C), while there was no difference between the wild-type and mutated anosmin-1-induced NLT migration (Fig. 3B,C). Taken together, the results suggested that the mutated amino acid did not impair the structure or chemomigration function of anosmin-1, indicating that this variation resulted in KS through another mechanism.

### A m.5800A>G variant was observed in the mitochondrial tRNA^cys^ gene (*MT-tRNA* ^*cys*^)

Although unknown *KAL1* mutations were possible, we hypothesized that mitochondrial genes might also affect anosmin-1 functions to some extent, as all the KS patients shared the same maternal mitochondrial genomes due to lack of offsprings of the patients, which meant no crisscross inheritance existed in the X-linked inherited KS family. Therefore, we screened for variations in the mitochondrial genomes of the matrilineal members.

**Figure 4:**
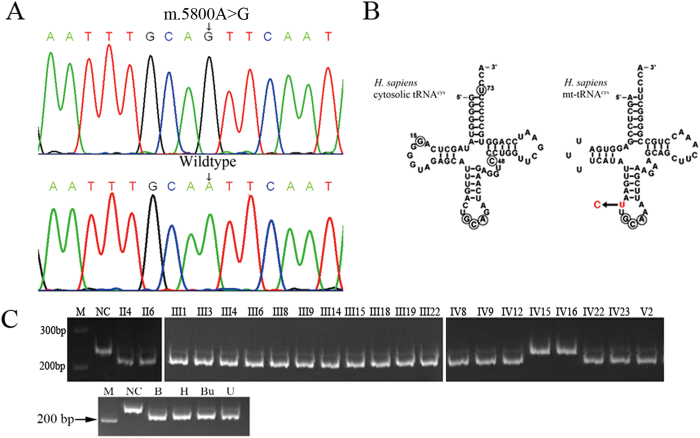
A newly occurred mtDNA variant (m.5800A>G) was observed in the matrilineal mitochondrial genome. (**A**) The sequencing chromatograph of the mitochondrial genome of the matrilineal members. At nucleotide 5800, all members carried a guanine, while this residue was an adenine in the mitochondrial genome of the normal control. (**B**) The adenine-to-guanine variant occurred on the 5′ of the mitochondrial tRNA^cys^ anticodon. (**C**) PCR-RFLP showed that this variant was nearly homoplasmic at the 5800 site; samples were taken from the hair roots of the matrilineal members and from different tissues of the index case. NC: normal control B: Blood; H: hair root; Bu: buccal mucosa; U: urine.

Thirty-three variants were identified in the matrilineal lineage, of which 32 variants were previously identified polymorphisms with no known consequences (Supp. Table S2). One variant, however, was a previously undescribed adenine-to-guanine transition at nucleotide 5800, which resided within the mitochondrial tRNA^cys^ gene (Fig. 4A). The adenine-to-guanine mutation occurred adjacent to the 5′ end of the mitochondrial tRNA^cys^ anticodon (Fig. 4B). This variant was found only on the matrilineal lineage in this family (Fig. 4C and Supp. Figure S2), it did not appear among the thousands of mitochondrial genomes previously sequenced^31^, and it was absent in 2,000 unrelated control individuals. PCR-RFLP revealed that this mutation was nearly homoplasmic in the different tissues of the index case and in the hair roots of all of the matrilineal members, regardless of their phenotypes (Fig. 4C). Although mitochondrial DNA at site 5800 is not very conserved among different species and human cytosolic tRNA^cys^ uses C at this site (Fig. 4B), nevertheless, it is highly conserved in human mitochondrial genome. Considering high mutation rate in the mitochondrial DNA, it may exert distinct roles in human mitochondrial metabolism to some extent.

### Perturbance in the mitochondrial cysteinyl-tRNA pathway affects the migration of the neuroendocrine GnRH cells through anosmin-1

To test the role of the mitochondrial cysteinyl-tRNA pathway in the anosmin-1-induced GnRH cell migration, we exposed NLT cells to conditioned media containing wild-type or mutated anosmin-1 secreted from 293T cells exogenously expressing mitochondrial cysteinyl-tRNA synthetase, CARS2, which is localized to the mitochondrion (Supp. Figure S5) and specifically catalyzes the addition of cysteine to the mitochondrial tRNA^cys^ or from cells in which CARS2 was knocked-down. We noticed that the signals of both wild-type and mutated anosmin-1-GFP were aggregated or dispersed after overexpression or knockdown of CARS2, respectively (Fig. 3A). Thus, it was suggested that the level of CARS2 might affect the synthesis or secretion of anosmin-1.

After overexpression or knockdown of CARS2, GFP-tagged wild-type and mutated anosmin-1 were expressed in 293T cells to produce conditioned media. The number of migrated NLT cells induced by wild-type anosmin-1 was significantly increased by 1.4-fold; on the contrary, treatment of 293T cells with si-CARS2 reduced anisom-1-induced GnRH cell migration (Fig. 3B,C). To our surprise, the mutant anosmin-1 from the CARS2 overexpressing cells remarkably decreased the migration rate to almost 50% compared to the wild-type medium, while there was no significant difference between the wild-type and mutant anosmin-1-induced GnRH cell motility in the CARS2 depletion conditions (Fig. 3B,C). It was possible that m.5800A>G variant in *tRNA*^*cys*^ might result in an *in vivo* condition paralleled with CARS2 overexpression. We further investigated whether defects in the mitochondrial cysteinyl-tRNA pathway affected GnRH neuron motility directly using *in vitro* wound healing assays. Additionally, wound closure in the mCARS2-transfected NLT cells was not significantly different than the control cells after scratching (Supp. Figure S6A and S6B). These results demonstrated that mitochondrial cysteinyl-tRNA pathway defects in the GnRH neurons may not impair the cell motility directly, but this pathway may play an important role in conditioned medium production, and thus, affected the chemomigration of GnRH neurons.

Then we hypothesized that the synergism of the variations in both mitochondrial tRNA^cys^ and anosmin-1 could repress GnRH cell migration. To address this hypothesis, EBV-transformed B cell lines were established from the whole blood of normal controls and KS patients to investigate whether the mutated mitochondrial tRNA^cys^ showed a similar phenomenon as CARS2 exogenous expression. As we expected, the conditioned media from the wild-type anosmin-1-transfected KS or control B cell lines, as well as the mutant anosmin-1-transfected control B cell lines, could induce the migration of NLT cells (Fig. 3D,E). However, the number of migrated cells was reduced more than 1.3-fold when the cells were treated with the medium from the B cell lines of KS patients containing the mutated anosmin-1 (Fig. 3D,E). Furthermore, there was no significant difference in the oxidative phosphorylation (ATP level), mitochondrial translation and structure of the EBV-transformed B cells from the control and KS patients (Supp. Figure S7A and S7B). The media from the wild-type and mutated anosmin-1-transfected control or KS B cell lines did not affect the growth of the NLT cells (Supp. Figure S7C). Taken together, these results showed that the synergistic effect of the mutation of the anosmin-1 and MT-tRNA^cys^ might cause the abnormal migration of the GnRH neurons.

### Knockdown of the *cars2* gene in *Danio rerio* resulted in ectopic migration of GnRH3 cells

**Figure 5:**
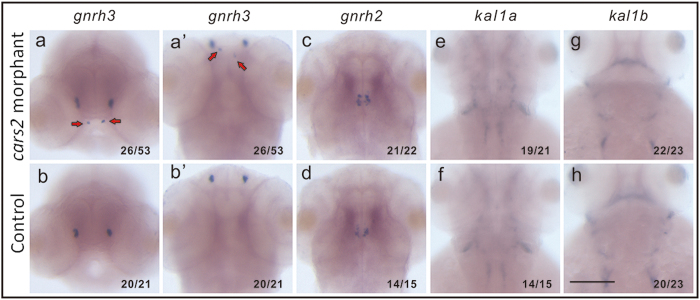
*cars2* gene knockdown with Morpholinos in *Danio rerio* affected the migration of GnRH3 neurons. Whole mount *in situ* hybridization of the control (b (b’),d,f,h) and *cars*2 morphants (a (a’),c,e,g) embryos with *gnrh3* (a (a’),b (b’)), *gnrh2* (c,d), *kal1a* (e,f), and *kal1b* (g,h) probes at 56 hpf. (a,b) front views, dorsal up; (a’,b’,c–h) dorsal view, anterior up. Results shown were representative figures from three independent experiments and the frequency of embryos with the indicated expression patterns were shown in the bottom left corner of each panel. Red arrows indicated the affected GnRH3 neurons by knockdown *cars2*. Scale bar: 0.1 mm.

To investigate whether the perturbance in mitochondrial cysteinyl-tRNA pathway could also affect GnRH neuron migration *in vivo*, zebrafish larvae were used to establish a *cars2* knockdown model. Zebrafish have two types of GnRHs, GnRH2 and GnRH3. Zebrafish GnRH3 neurons are distributed at both the preoptic area-hypothalamus (POA-hypo) and the olfactory bulb–terminal nerve (OB-TN) in the brain, which resemble the mammalian hypophysiotropic GnRH1 neurons and are important for gonadal development and reproduction, while zebrafish GnRH2 are located in the midbrain tegmentum^32^,^33^. Zebrafish GnRH3 neurons also originate from the olfactory bulb region and migrate posteriorly to the hypothalamus. We used morpholinos (MOs) to knockdown zebrafish *cars2* and then employed *in situ* hybridization assay to detect GnRH3 neurons, GnRH2 neurons, and expression of *kal1a* and *kal1b* (co-orthologs of mammalian *KAL1*)^34^. As shown in Supp. Figure S8, cars2-i2e3 MO could effectively block splicing of *cars2*. Compared to the control (Fig. 5b,b’), the results showed that in cars2-morphants, GnRH3 neurons but not GnRH2 neurons displayed ectopic migration outside of the olfactory bulb region (Fig. 5a,a’). In addition, knocking down *cars2* had no effects on expression of *kal1a* and *kal1b* (Fig. 5e–h). These results clearly suggest that *cars2* is essential for the migration of zebrafish GnRH3 neurons *in vivo*.

### *MT-tRNA* ^*cys*^ variant might be prevalent in CHH/KS individuals

We then aimed to determine the frequencies of the *KAL1* and *MT-tRNA*^*cys*^ gene variants in CHH/KS population. We screened the available blood sample of 23 sporadic CHH patients and 15 sporadic or familial KS individuals. Interestingly, we identified one CHH patient carried heteroplasmic m.5800A>G variant in *MT-tRNA*^*cys*^ gene. No c.146G>T variant in *KAL1* gene was detected. Due to the importance of CARS2 involved in the mitochondrial metabolism, no mutation was found in our limited patient’s samples. More efforts needed to put in analyzing the variant scope and frequency of *MT-tRNA*^*cys*^ gene or *CARS2* in a large scale of CHH/KS individuals.

## Discussion

Kallmann syndrome is a human genetic disease that is characterized by impaired cell migration and axon elongation. It has been widely reported that both olfactory neuron axon elongation and GnRH synthesizing neuron migration are defective in KS patients^2^. Anosmin-1 is an extracellular matrix glycoprotein that is thought to be important for the targeting of embryonic olfactory nerve fibers to the presumptive olfactory bulbs. This protein contains an N-terminal, cysteine-rich domain (Cys-box) with five putative disulfide bridges, a whey acidic protein-like (WAP) domain with four disulfide bridges, and four fibronectin-like type III (FnIII) domains^35^. Mutation analysis has revealed several variants in the *KAL1* gene in patients with KS. Here, we characterized a novel variant in *KAL1* (c.146G>T (p.Cys49Phe)) in a large Han Chinese family with inherited KS. The mutation site was located in the Cys-box region of anosmin-1. The Cysteine-rich domains in anosmin-1 are often found in the epidermal growth factor receptor (EGFR) superfamily as the second and fourth of a four-domain structure that constitutes a receptor tyrosine kinase family involved in the interaction of FGFR1^35^,^36^ and signal transduction^35^,^37^. However, the mutant anosmin-1 exhibited no difference in predicated 3D structure, subcellular localization in transfected cells, or ability to induce GnRH neuron migration in an *in vitro* assay, compared to wild-type anosmin-1, indicating that the mutation may not impair the cellular secretion or the chemomigration function of anosmin-1. Notably, at least five mutations previously identified in KS patients affect cysteine residues forming disulphide bonds in the whey acidic protein-like domain of the anosmin-1 (C134G, C163R, C163Y, C164del, and C172R)^38^,^39^,^40^,^41^, but mutations affecting a cysteine residue in the N-terminal, cysteine-rich region of the protein had not yet been reported.

In X-linked inherited KS families, male patients are infertile, so all of the consanguineous male KS patients shared the same maternal mitochondrial genomes. Certain mitochondrial backgrounds may modulate the susceptibility to disease, and this may be linked to variations in oxygen consumption, the efficiency of electron transport, ATP generation, and reactive oxygen species (ROS) production. Therefore, the mitochondrial genomes of this KS family were scanned, and a novel mitochondrial *tRNA*^*cys*^ mutation (m.5800A>G) was identified. The anticodon loop results in a sharp turn in the phosphodiester backbone, allowing the presentation of the anticodon to its cognate mRNA codon in the ribosome^42^,^43^. This turn is stabilized by a hydrogen bond between the amino group of the conserved uridine and the phosphate backbone of the third base of the anticodon. Cytidine lacks this amino group and cannot form this hydrogen bond. Biochemical studies with anticodon stem-loop analogs of tRNAs have been performed and have indicated that the substitution of cytidine for uridine at this position markedly impaired ribosome binding^44^, providing evidence of the functional importance of this mutation.

Mitochondria are the energy powerhouse of the cell and are present in almost all mammalian cells. Compared to the nuclear genome, the mitochondrial genome has a 10- to 17-fold higher mutation rate, and more than 200 variants associated with pathogenicity have been identified within the human mitochondrial genome^45^. Although mitochondrial tRNA sequences comprise only 10% of the mitochondrial genome, more than 50% of the characterized pathology-related mtDNA mutations are concentrated within the tRNA genes^45^. Therefore, studies on mitochondrial tRNA gene variants provide an interesting opportunity to explore the interface between genetic and biochemical factors that lead to pathogenesis. Because of the fundamental role served by the anticodon, it is understandable that there is an almost total absence of mutations in the anticodon triplet. Therefore, most of the mutations in the anticodon loop are adjacent to these anticodons (www.tRNA.uni-bayreuth.de)^46^. However, information concerning the factors that determine the pathogenicity of specific mitochondrial tRNA mutations is very limited. A deleterious mutation on a fundamental mitochondrial gene such as tRNA^cys^ would be expected to affect other tissues and organs and to cause more widespread clinical disorder and not just isolated KS. However, we measured several mitochondrial toxicity markers, including the oxidative phosphorylation (ATP level), mitochondrial membrane potential levels and cell survival in EBV-transformed B cells from control and KS patients. And there was no significant difference. Also, no other mitochondrial related disorder has been found in patients from this family. Thus, it suggests that this *tRNA*^*cys*^ m.5800A>G variant may not seriously impair mitochondrial metabolism except for synergistic effect on the migration of GnRH neurons with *KAL1* variant.

Aminoacyl-tRNA synthetases are evolutionarily conserved enzymes that attach a specific amino acid to the end of its cognate tRNA^47^. Aminoacyl-tRNA synthetase activity is required in the cytoplasm and in the mitochondria for the translation of nuclear and mitochondrial genes, respectively. In mammals, most cytoplasmic and mitochondrial tRNA synthetases are encoded by different genes. Recently, only two mitochondrial tRNA synthetases have been found to harbor mutations which have effects on fertility. These mutations include the c.598C>G and c.1102G>T mutations in HARS2, which encodes a histidyl-tRNA synthetase that is predicted to function in the mitochondria, and the c.1565C>A mutation in LARS2, which encodes mitochondrial leucyl-tRNA synthetase^48^,^49^,^50^. Mutations in both of these genes lead to Perrault syndrome, which highlights the critical role of the mitochondria in the maintenance of ovarian function and hearing. For the first time, we found that defects in the mitochondrial cysteinyl-tRNA synthetase CARS2 could also cause altered physiological functions. Anomalous GnRH cell migration was observed in *cars2-*deficient zebrafish. It is intriguing to consider CARS2 or genes involved in the mitochondrial cysteinyl-tRNA pathway are candidates for biochemical and genetic screenings of KS patients.

Given the complexity of mitochondrial genetics and biochemistry, the exact mechanisms by which biochemical cascades can be dramatically affected by mitochondrial tRNA mutations still remain uncharacterized^45^. In the present study, disturbing the mitochondrial cysteinyl-tRNA pathway could affect the function of anosmin-1, but not its expression. However, as a secreted protein, anosmin-1 forms a disulfide bond and folds in the endoplasmic reticulum^30^,^35^. More efforts need to be carried out to characterize the molecular mechanism.

In summary, two novel variants, *KAL1* (c.146G>T (p.Cys49Phe)) and mitochondrial tRNA^cys^ (m.5800A>G), were identified in a large Han Chinese family with inherited KS. Our findings demonstrated that the disturbance of the mitochondrial cysteinyl-tRNA pathway could impair GnRH cell migration *in vitro* and *in vivo*. These results might open new avenues to better understand the contributions of nucleo-cytoplasmic gene interactions to the genetic etiology of Kallmann syndrome.

## Patients, Materials and Methods

### Subjects

As a part of the genetic screening program for male infertility in Chinese patients, the proband suffering from KS was found at Shanghai Institute of Planned Parenthood Research/WHO Collaborating Center for Research in Human Reproduction and was diagnosed at First Affiliated Hospital of Anhui Medical University. All patients in this family were interviewed and evaluated to identify both the personal and medical histories of reproduction and other clinical abnormalities. Written, informed consent, which conformed to the tenets of the Declaration of Helsinki, was obtained from each participant prior to the study. The ethics committee of University of Science and Technology of China (USTC) approved this study.

### Cell migration assays

Transwell assays were performed in 12-well polycarbonate transwell migration chambers with 8-μm pores (Corning), according to the instructions. Briefly, the transwell plates were coated with Matrigel Basement Matrix (BD Biosciences). The lower compartment of the chamber was loaded with 700 μl of control medium or medium from 293T cells transfected with the following vectors: pcDNA/CARS2, pEGFP-N1/KAL1, or KAL1 G146T. The NLT cells were grown in complete medium until they reached subconfluence, and 4 × 10^4^ cells were seeded in the upper surface of the Boyden chamber in medium containing 1% serum. After 24 h, the cells were stained with crystal violet. The number of adherent cells was counted after the non-migrated cells were scraped off of the upper surface of the porous filter. The experiments were performed at least in triplicate.

### Zebrafish cars2 morpholinos and microinjection

Wild-type AB strain (*Danio rerios*) were bred and maintained at the Soochow University Zebrafish Facility according to standard protocols. Zebrafish were maintained on a 14 hour/10 hour light:dark cycle. The embryos were collected in the morning from group crosses containing two males and two females. All animal experiments were performed in accordance with the guidelines and regulations of the Soochow University. Morpholinos (MOs), which are modified oligonucleotides that interfere with mRNA translation, were used to knockdown the zebrafish *cars2* gene. All of the following MOs were synthesized by Gene-Tools LLC: Control MO: 5′- CCT CTT ACC TCA GTT ACA ATT TAT A -3′; *cars2* MO: 5′- ATG AAC TGC ACA GAA GAA AGA GGA T -3′. Morpholinos (4 ng) were injected into one-cell embryos as previously described^51^.

### Statistical analyses

All experiments were performed in triplicate and were repeated at least three times. The data are presented as the mean ± SEM. Student’s t-test was used to compare the data between two groups (two-tailed, unequal variance). Analysis of variance was conducted to compare the data of more than two groups using the Chi-square test or a one-way or two-way ANOVA. The differences were considered significant when P < 0.05.

## Additional Information

**How to cite this article**: Wang, F. *et al.* Point mutations in *KAL1* and the mitochondrial gene *MT-tRNA*^cys^ synergize to produce Kallmann syndrome phenotype. *Sci. Rep.* **5**, 13050; doi: 10.1038/srep13050 (2015).

## Supplementary Material

Supplementary Information
