# Supplementary material for: Point mutations in KAL1 and the mitochondrial gene MT-tRNAcys synergize to produce Kallmann syndrome phenotype
Source: Sci Rep. 2015 Aug 17;5:13050. doi: 10.1038/srep13050 (PMC4642522; doi:10.1038/srep13050)
Supplement: Supplementary Information [file srep13050-s1.pdf]

Point mutations in *KAL1* and the mitochondrial gene *MT-tRNA^cys^* synergize to produce Kallmann syndrome phenotype

Fei Wang[1†], Guo-dong Huang[2,3†], Hui Tian[1], Ying-bin Zhong[2,3], Hui-juan Shi[4], Zheng Li[5], Xian-sheng Zhang[6], Han Wang[2,3]*, Fei Sun[1]*

Supplementary data

The p.Cys49Phe variation in anosmin-1 did not significantly affect the protein structure.

Based on the degree of amino acid conservation in the sequence alignments derived from closely related sequences, this mutation was predicted to be benign by the PolyPen-2 program (Score: 0.35), while is shown to damaging by the SIFT program (SIFT score: 0.01). The difference between two prediction results might be due to *KAL1* gene's particularity. For example, there were no homologs found in mouse and rat genomes now. Furthermore, as shown in Supp. Figure S3, the 3D structure of the anosmin-1 protein, which was determined using the protein homology/analogy recognition engine (Phyre2) computational tool (http://www.sbg.bio.ic.ac.uk/phyre2), showed that the mutated residue of anosmin-1 was in the cysteine box region, which might be involved in its interaction with FGFR1. However, the mutant anosmin-1 shared the same 3D structure and electrostatic potential as the wild-type anosmin-1 (Supp. Figure S3).

Supplementary methods

Protein structure imitation and pathogenicity Prediction

To analyze the possible impacts of the amino acid substitution caused by p.Cys49Phe mutation on the three-dimensional protein structures and its consequence on protein functions, we used the PolyPhen program (http://genetics.bwh.harvard.edu/pph/) and the SIFT program (http://sift.jcvi.org/www/SIFT_enst_submit.html), which allow to predict whether an amino acid change is likely to be deleterious to protein function. Secondary structure of wild-type and mutated anosmin-1 had been predicted using PHYRE2 (http://www.sbg.bio.ic.ac.uk/phyre2).

Mutation Screening

Different tissues were collected from the family members, and genomic DNA was extracted using standard methods. Fourteen exons of the *KAL1* gene were amplified and analyzed by Sanger sequencing (Invitrogen, USA). For analysis of the entire mitochondrial genome, eight overlapping fragments were amplified by PCR using 8 sets of primers; these fragments were purified and subsequently analyzed by direct sequencing (Invitrogen, USA). The assembled sequence was compared to the revised consensus Cambridge sequence (rCRS, GenBank accession number: NC-012920). PCR-based restriction fragment length polymorphism (PCR-RFLP) analyses were used to screen for single nucleotide polymorphisms (SNPs) in the *KAL1* gene and the mitochondrial DNA site at position 5800 using standard methods. The uniqueness of the *KAL1* and mtDNA variations in this family was confirmed by an exhaustive search in the relevant databases (dbSNP, 1000 Genome Project, NHLBI Exome Variant Server and human mitochondrial database) and by screening at least 2,000 control individuals of the same origin with no history of KS. Nucleotide numbering used +1 as the A of the ATG translation initiation codon in the reference sequence, with the initiation codon as codon 1. For mtDNA, nucleotide numbering referred to rCRS.

DNA Constructs

GFP-tagged anosmin-1 was constructed by inserting the coding region of human *KAL1* into the MCS of the pEGFP-N1 vector, whereas c.146G>T mutated *KAL1* was generated using a PCR-based site-directed mutagenesis method. The vectors expressing human and mouse CARS2 were constructed in the pcDNA3.1+ vector. The siRNAs against CARS2 were obtained from Santa Cruz Biotechnology, Inc. (Dallas, Texas, USA).

Establishment of the EBV-transformed B-cell lines

Peripheral blood was obtained from the patients and the control, and 1-2 ml of whole blood was

transferred into 15-ml conical centrifuge tubes. A Ficoll-Hypaque gradient was used to separate the mononuclear cells, and the cells were resuspended in transformation medium, which consisted of RPMI-1640 medium containing harvested EBV stock, 15% fetal calf serum, and 0.03 mg/ml cyclosporin A. The suspension cultures were incubated 7 to 10 days in 25-cm^2 tissue culture flasks and were periodically fed by replacing one third of the medium. The transformed B-cell lines emerged after approximately 6 weeks, after which they rapidly became the only viable elements in the cultures.

Cell culture, cell proliferation, and immunofluorescence

Mouse GnRH neuronal NLT cells were kindly provided by Xiao-juan Zhu (Northeast Normal University, China) and were cultured in DMEM supplemented with 10% fetal bovine serum (FBS), 100 U/ml penicillin and 100 μg/ml streptomycin (Invitrogen, Carlsbad, CA); the cells were incubated under standard conditions (5% CO_2, 37 ℃). To examine the subcellular localization of anosmin-1, HEK293T cells were transfected with pEGFP-N1/KAL1 or KAL1 G146T for 48 h. The cells were then fixed with 4% paraformaldehyde for 30 min, and the nuclei were stained with Hoechst 333342 (Sigma). The fluorescent signals were examined under a Nikon epifluorescence microscope (Nikon Eclipse 80i; Nikon, Tokyo, Japan).

Whole-mount in situ hybridization

Embryos were fixed in phosphate-buffered 4% paraformaldehyde. Whole-mount *in situ* hybridization was performed. cDNA fragments for the *gnrh2*, *gnrh3*, *kal1a*, and *kal1b* sequences were cloned into the pESAY-T3 vector (Transgen) and were used to synthesize antisense RNA probes with digoxigenin-UTP.

Wound healing assay

NLT cells were incubated in 12-well plates and wounded in the confluent monolayer using a thick plastic cell scraper (Corning). Then, the cells were rinsed two times with PBS and fresh culture medium was added. Cells were captured under a microscope (Eclipse Ti-S, Nikon) at 0, 2, 4, 6, 9, 12 and 24 hours after scrape. Total distance migrated by cells in indicated time was determined as the difference of the cell front relative to the 0 hour timepoint. Experiments were repeated three times, with three distance measurements per field of view.

Supplementary figures

Supp. Figure S1 X chromosome region Xp22.32 was closely linked to the KS phenotype.

Two polymorphic STR markers on X chromosome region Xp22.32, DXS9895 and DXS9906, flanking *KAL1* gene were used for linkage analysis. The genotypes of the family members were shown by numbers. The haplotype ‘3 4’ was closely linked to the KS phenotype.

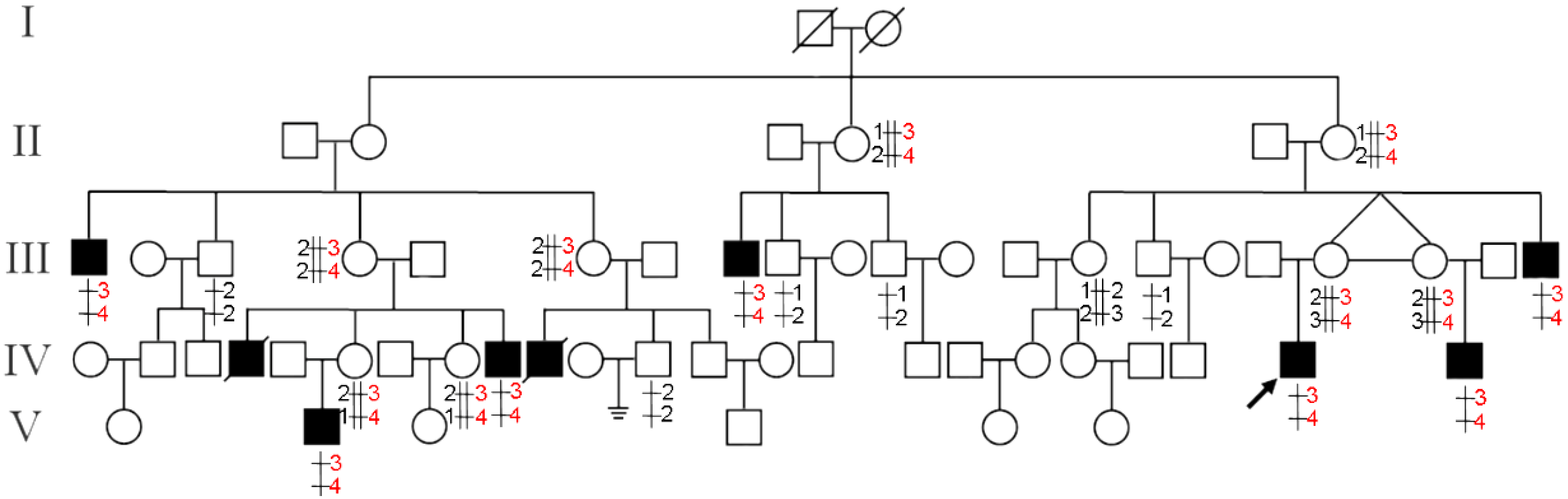

Supp. Figure S2 Genotypic profiling of the KS family.

Genotyping of *KAL1* c.146G>T variant and mitochondrial DNA m.5800A>G variant was conducted by PCR-RFLP. +: *KAL1* wild type; T: *KAL1* c.146G>T; 5800A: mtDNA wildtype; 5800G: m.5800A>G

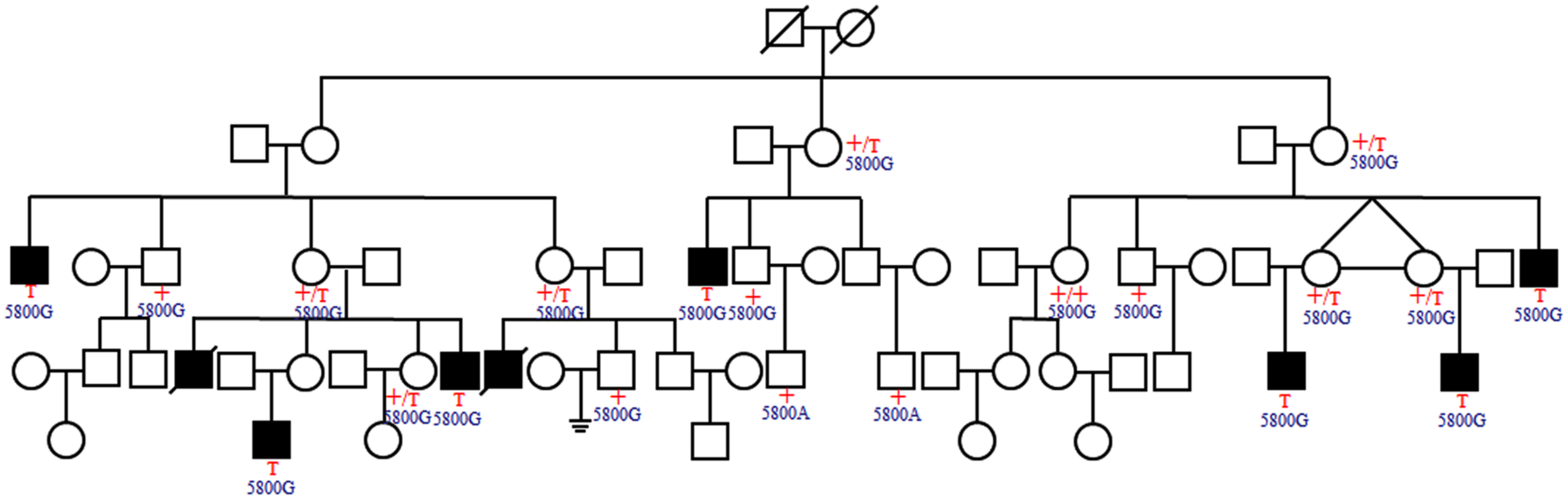

Supp. Figure S3 Prediction of structures of anosmin-1.

Structure prediction of wild type (up panel) and mutant type anosmin-1 (down panel). The sticks of mutated residue was circled. Electrostatic potential mapped onto the molecular surface of anosmin-1 and mutant anosmin-1 with the two bound potentials were contoured from -5 (red) to +5 kT/e (blue).

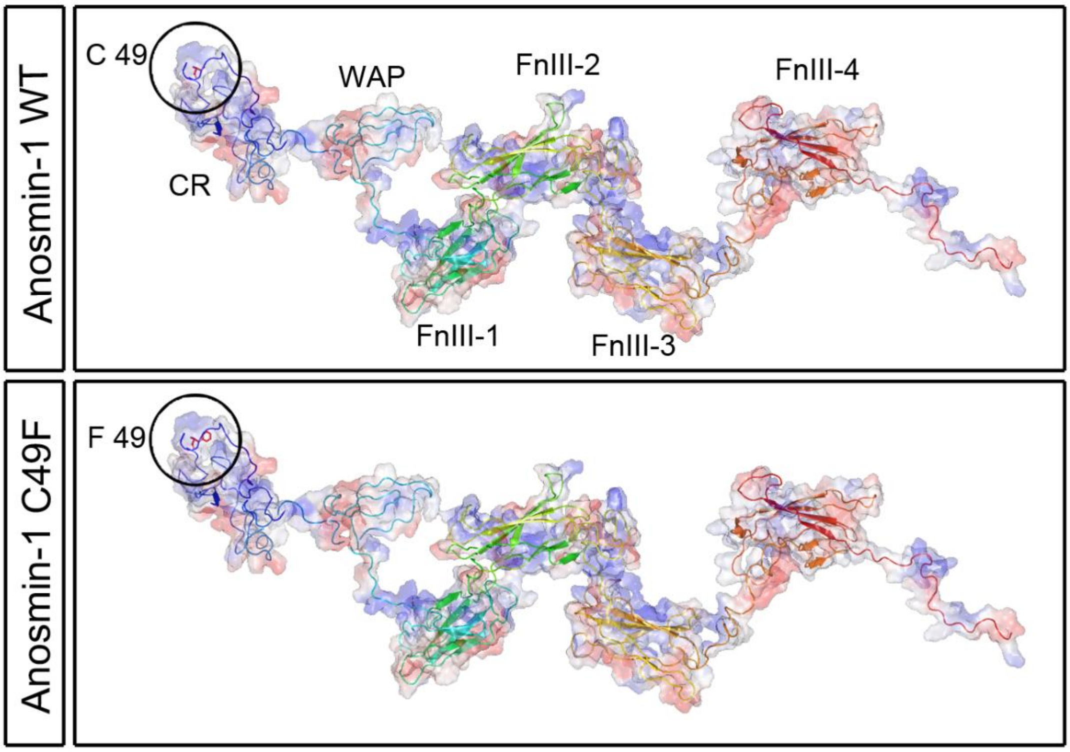

Supp. Figure S4 The nonsynonymous amino acid substitution in anosmin-1 might not affect the subcellular localization and expression of anosmin-1. (A and B). Both the anosmin-1-GFP and mutated anosmin-1-GFP proteins were highly expressed in 293T cells (A) and localized in the endoplasmic reticulum (ER) and in the Golgi region (B). GM-130, a cis-Golgi matrix protein, as the Golgi complex marker to demonstrate the localization of anosmin-1 in the cytoplasm. Red Arrow indicated anosmin-1-GFP accumulated in ER and Golgi region. Red arrow heads indicated anosmin-1-GFP localized in Golgi region. When 293T cells were transfected with wild type or mutated *KAL1* overexpression vectors for 24 h and treated with 10 μg/ml brefeldin A, a protein transport inhibitor which blocks ER-Golgi membrane trafficking, for 30min, accumulations of both wild type and mutated anosmin-1-GFP were detected in the cytoplasmic region (A). Blue, Hoechst; green, anosmin-1-GFP; Red, GM-130. Scale bar: 50 μm. (C and D). Western blotting analysis of expression level of anosmin-1-GFP protein, produced by transfected 293T cells (down panel) and presented in their culture media conditioned for 16–18 h (up panel) (C). The results were also shown no significant difference between them (D).

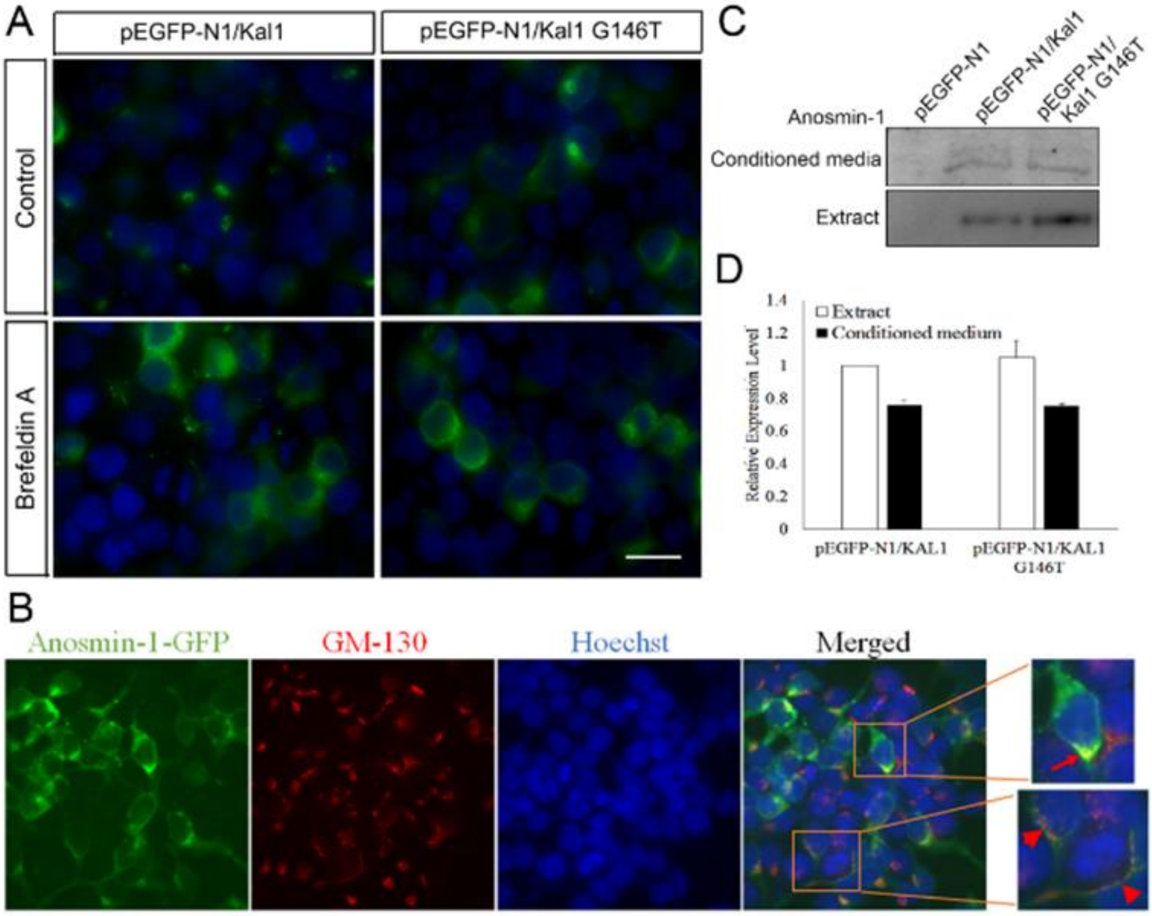

Supp. Figure S5 CARS2 was localized to the mitochondrion. (A). The phylogeny tree of CARS2 proteins with human CARS protein as an out-group. The tree was constructed by the neighbor-joining method with MEGA5.2. Hs, *Homo sapiens*, Mm, *Mus musculus*, Dr, *Danio rerio*, Dm, *Drosophila melanogaster*, Rn, *Rattus norvegicus*, Pt, *Pan troglodytes*, Xl, *Xenopus laevis*. (B). CARS2 was a mitochondrial protein. The 293T cells were transfected with GFP-tagged CARS2 and purified mitochondria were isolated by standard methods. Western blotting analysis indicated the distribution of CARS2 between cytoplasm and mitochondrion . Cytoplasmic protein GAPDH was used as the control.

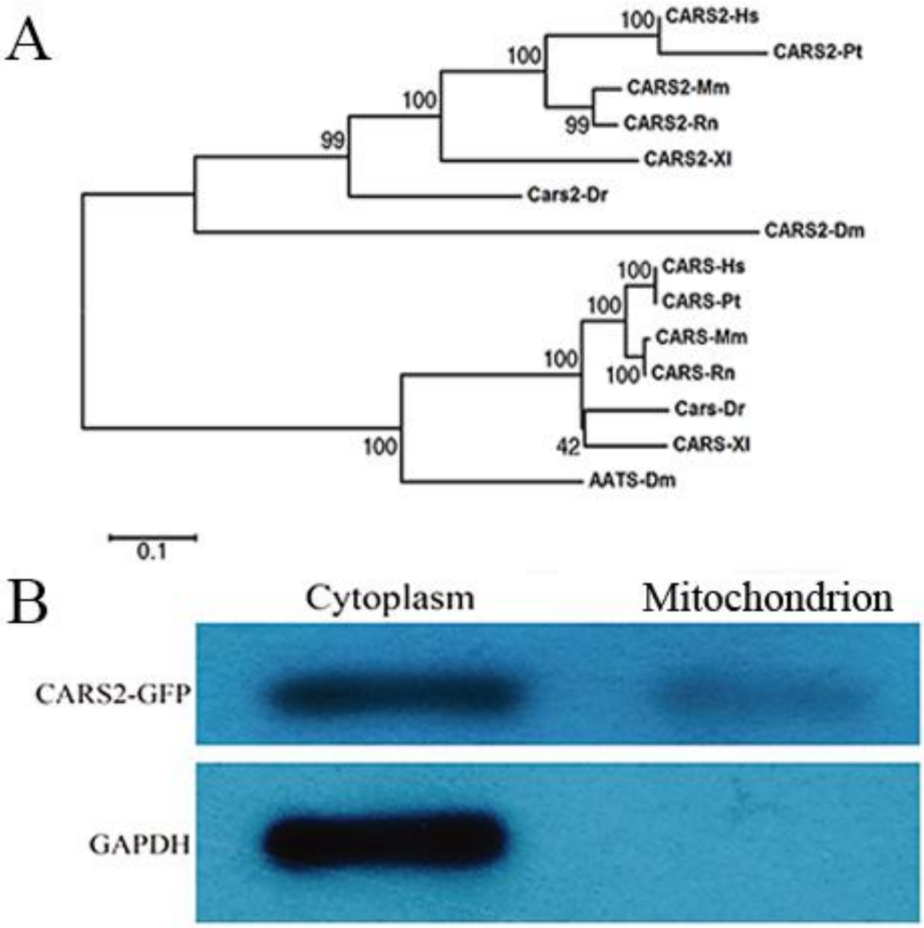

Supp. Figure S6 Overexpression of CARS2 did not affect the migration distance of NTL cells. (A). Serum starvation blocked NLT cells motility. Addition of serum induced robust migration of NLT cells that were transfected with pcDNA3.1 or pcDNA3.1/CARS2. Scale bar：50 µm. (B). Quantification shows no significant difference in the migration distance of CARS2 overexpressed cells (●) compared with cells transfected with control vectors (■).

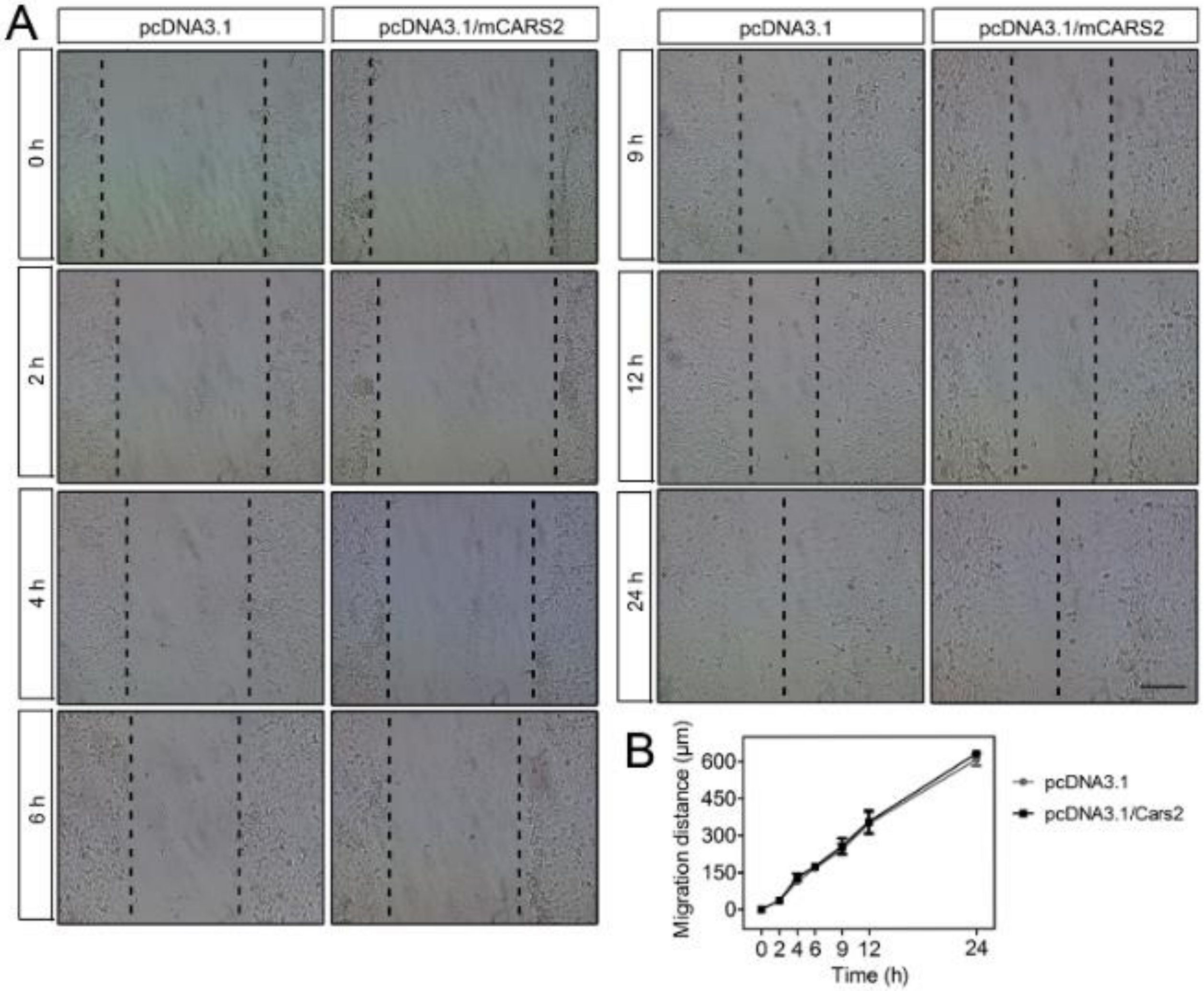

Supp. Figure S7 ATP levels, mitochondrial translation, structure and cell growth were not affected in KS patient.

(A). ATP level of B cell lines established from KS patients was similar as B cell lines from controls. (B). Expression levels of mitochondrion-encoded and nuclear-encoded mitochondrial proteins were not affected in B cell lines of KS patients. CYTB, a mitochondrion-encoded protein, is a component of the ubiquinol-cytochrome c reductase complex (complex III or cytochrome b-c1 complex), which is a respiratory chain that generates an electrochemical potential coupled to ATP synthesis. Mitofusin-1 (MFN1, NP_284941.2), a nuclear-encoded mitochondrial essential transmembrane GTPase, which mediates mitochondrial fusion. Western blotting analysis of expression level of B cell lines established from KS patients was similar as B cell lines from controls.

(C). The media from wild type and mutated anosmin-1 transfected control or KS B cell lines did not affect the growth of NLT cells. B cell lines from control or KS patients were transfected with pEGFP-N1, pEGFP-N1 KAL1 or pEGFP-N1 KAL1 G146T for 24 h and different groups of media were collected to treat 293T cells. After 24 h, cell growth was detected by CCK-8 assay.

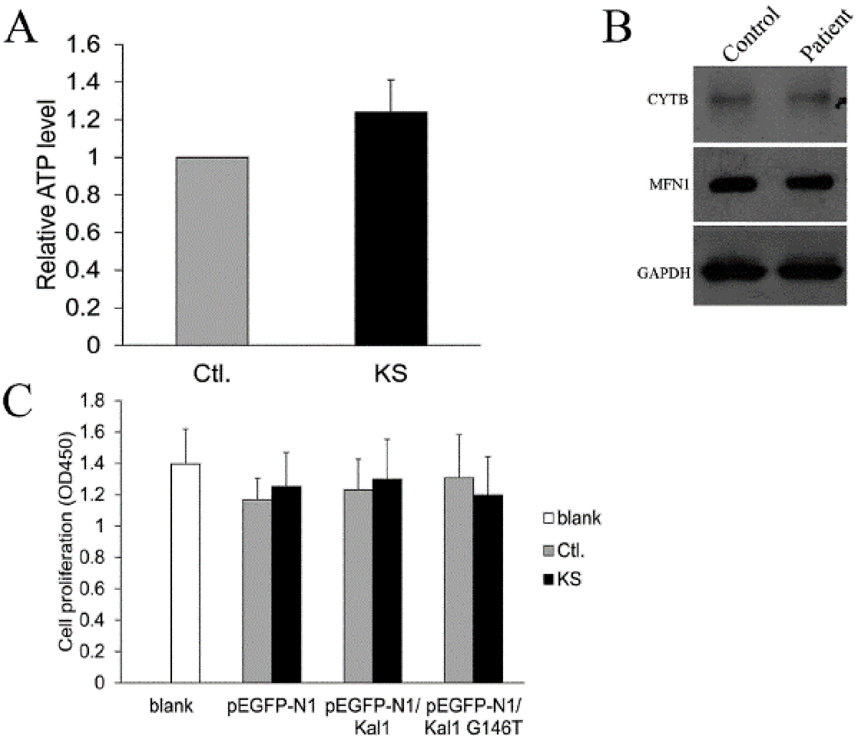

Supp. Figure S8 cars2-i2e3 splicing blocking MOs efficiently knocked-down *cars2* in zebrafish.

(A). The efficacy of cars2-i2e3 splicing blocking MO. One-cell stage of WT zebrafish embryos were injected with 8 ng control MO (cMO) and 2 , 4 , or 8 ng cars2-i2e3 MO.

(B). Toxicities caused by cars2-i2e3 MO in zebrafish embryos. 4 ng of cMO or cars2-i2e3 MO was injected into 1-cell stage WT embryos. One day after injection, normal, deformed, unfertilized and dead embryos were recorded. The results indicated three independent experiments.

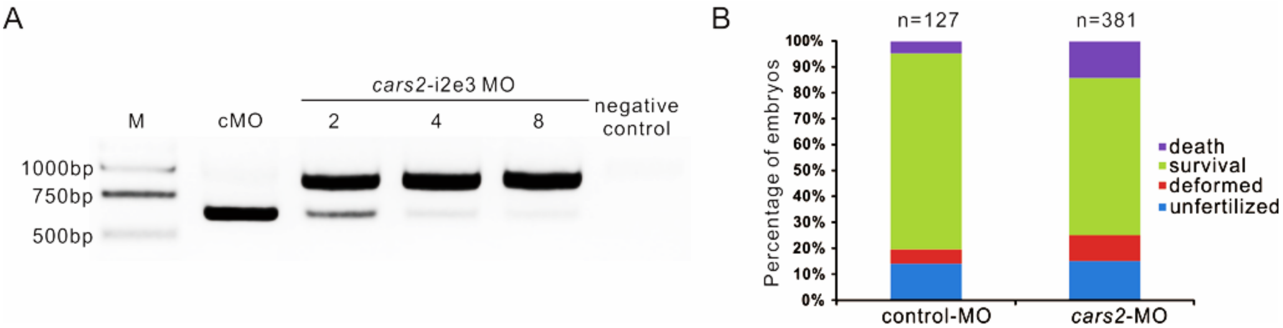

Supplementary Tables

Supp. Table S1 Clinical and hormonal characteristics of the available members with KS

Subject	III1	III8	III21	IV9	IV22	IV23	V2 [a]
age at diagnosis (years)	>50	>50	>50	30	29	27	14
Height (cm)/ Weight(Kg)	>170/74	>170/76	185/86	165/55	170/60	185/75	150/36
Reproductive phenotype at diagnosis	Hypogonadism<br>No offspring	Hypogonadism<br>No offspring	Hypogonadism<br>No offspring	Hypogonadism<br>Penis: 4 cm<br>Mean TV: 3 ml<br>Right cryptorchidism	Hypogonadism<br>Penis: 4 cm<br>Mean TV: 2.5 ml	Hypogonadism<br>Penis: 4.5 cm<br>Mean TV: 3 ml	Hypogonadism? [a]<br>Delayed puberty<br>Penis: 3 cm<br>Mean TV: 2.5 ml
Sense of smell	Hyposmia	Hyposmia	anosmia	Hyposmia	anosmia	anosmia	Hyposmia
Testosterone[b] (nmol/l)	ND	ND	ND	0.20	0.34	0.51	NS
FSH[b] (IU/l) basal	ND	ND	ND	0.52	0.88	1.52	NS
LH[b] (IU/l) basal	ND	ND	ND	0.1	0.1	1.2	NS
Other dysfunctions	Hypertension<br>Dyslipidemia<br>Hyperglyceria	Hypertension<br>Dyslipidemia<br>Hyperglyceria	Hypertension<br>Dyslipidemia<br>Hyperglyceria	No	No	Hearing loss[c]	No

ND: not determined; NS: not sure

[a]Receiving hormone replacement at the time of diagnosis

[b]Normal range in adults, basal LH: 1.7-8.6 IU/l; basal FSH: 1.5-12.4 IU/l; testosterone in men: 9.9-27.8 nmol/liter

[c]Aminoglycoside-induced hearing loss

Supp. Table S2 Mitochondrial DNA variants in mitochondrial genome of the family member III-18

Variation	Type	AA position	AA change	Locus name
73	A>G			dloop
249del				dloop
263	A>G			dloop
315+c				dloop
523-524del				dloop
750	A>G			rRNA12s
1005	T>C			rRNA12s
1438	A>G			rRNA12s
1824	T>C			rRNA16s
2706	A>G			rRNA17s
3970	C>T	222	Syn(Leu)	nd1
4769	A>G	100	Syn(Met)	nd2
4811	A>G	114	Syn(Trp)	nd2
5800	A>G			$tRNA^{cys}$
6392	T>C	163	Syn(Asn)	co1
7028	C>T	375	Syn(Ala)	co1
7828	A>G	81	Syn(Leu)	co2
8860	A>G	112	Thr>Ala	atp6
8865	G>A	113	Syn(Val)	atp6
10310	G>A	84	Syn(Leu)	nd3
10535	T>C	22	Syn(Tyr)	nd4l
10586	G>A	39	Syn(Ser)	nd4l
11719	G> A	320	Syn(Gly)	nd4l
12338	T>C	1	Met>Thr	nd5
13708	G>A	458	Ala>Thr	nd5
13879	T>C	515	Ser>Thr	nd5
13928	G>C	531	Ser>Thr	nd5
14766	C>T	7	Thr>Ile	cytb
15326	A>G	194	Thr>Ala	cytb
16092	T>A			dloop
16245	C>T			dloop
16291	C>T			dloop
16304	T>C			dloop

*AA: amino acid   Syn: Synonymous variant

*Nucleotide numbering refers to the revised consensus Cambridge sequence (rCRS, GenBank accession number: NC-012920).
